# Structural insights into diverse modes of ICAM-1 binding by *Plasmodium falciparum*-infected erythrocytes

**DOI:** 10.1073/pnas.1911900116

**Published:** 2019-09-16

**Authors:** Frank Lennartz, Cameron Smith, Alister G. Craig, Matthew K. Higgins

**Affiliations:** ^a^Department of Biochemistry, University of Oxford, OX1 3QU Oxford, United Kingdom;; ^b^Liverpool School of Tropical Medicine, L3 5QA Liverpool, United Kingdom

**Keywords:** malaria, PfEMP1, ICAM-1, cytoadhesion

## Abstract

A major determinant of pathogenicity in malaria caused by *Plasmodium falciparum* is the adhesion of parasite-infected erythrocytes to the vasculature or tissues of infected individuals. This occludes blood flow, leads to inflammation, and increases parasitemia by reducing spleen-mediated clearance of the parasite. This adhesion is mediated by PfEMP1, a multivariant family of around 60 proteins per parasite genome which interact with specific host receptors. One of the most common of these receptors is intracellular adhesion molecule-1 (ICAM-1), which is bound by 2 distinct groups of PfEMP1, A-type and B or C (BC)-type. Here, we present the structure of a domain from a B-type PfEMP1 bound to ICAM-1, revealing a complex binding site. Comparison with the existing structure of an A-type PfEMP1 bound to ICAM-1 shows that the 2 complexes share a globally similar architecture. However, while the A-type PfEMP1 bind ICAM-1 through a highly conserved binding surface, the BC-type PfEMP1 use a binding site that is more diverse in sequence, similar to how PfEMP1 interact with other human receptors. We also show that A- and BC-type PfEMP1 present ICAM-1 at different angles, perhaps influencing the ability of neighboring PfEMP1 domains to bind additional receptors. This illustrates the deep diversity of the PfEMP1 and demonstrates how variations in a single domain architecture can modulate binding to a specific ligand to control function and facilitate immune evasion.

## Significance

Malaria is one of the deadliest infectious diseases to affect humans, causing over 200 million cases and hundreds of thousands of deaths annually. Its fatal symptoms occur when parasites cause infected human red blood cells to stick to human tissue surfaces, blocking blood flow and causing inflammation. This stickiness is caused by parasite PfEMP1 proteins, which interact with different human receptors, such as ICAM-1. In this paper, we demonstrate how PfEMP1 proteins bind to ICAM-1. We find that this can happen in 2 different but related ways, perhaps influencing which additional receptors PfEMP1 can bind. We show how the parasite can adapt to allow it to stick tightly, while reducing the chance that it is detected and destroyed.

Despite ongoing efforts to reduce global disease burden, malaria is still one of the world’s most prominent diseases, with an estimated 219 million cases each year (1). The symptoms occur as *Plasmodium* parasites divide within red blood cells of infected individuals. *Plasmodium falciparum*, the cause of the deadliest form of human malaria, invades and replicates within mature erythrocytes. This intracellular habitat reduces its susceptibility to detection by the mammalian immune system but makes it vulnerable to splenic clearance. However, the parasite also displays members of variant protein families on the surface of infected erythrocytes, including the multidomain *Plasmodium falciparum* erythrocyte membrane proteins 1 (PfEMP1). These PfEMP1 cause infected erythrocytes to adhere to the surfaces of blood vessels and tissues, removing them from circulation and protecting the parasite within from spleen-mediated destruction (2).

PfEMP1 have evolved under conflicting selection pressures. On one hand, they have diversified into a large protein family to evade immune clearance, with around 60 antigenically distinct members encoded in each parasite genome (3, 4). Since only a single PfEMP1 is usually expressed in each infected erythrocyte, this allows for antigenic switching, enabling the population to survive as cells displaying the previous PfEMP1 variant are detected and destroyed (5). On the other hand, PfEMP1 have maintained their capacity to bind specific human cell surface proteins, often allowing them to continue to mediate adhesion to human cell and tissue surfaces even after switching to a different PfEMP1 variant. Based on their chromosomal location, the majority of PfEMP1 are categorized in 3 major groups: A, B, and C (6, 7). Depending on the receptor to which they bind, expression of certain groups of PfEMP1 correlates with different symptoms and final outcomes of malaria episodes, with group A PfEMP1 associated with severe malaria, while the other groups tend to be linked with mild malaria, although the situation is less clear for group B PfEMP1 (8–10).

PfEMP1 bind to a variety of human ligands (11, 12), the most common of which are cluster of differentiation 36 (CD36), endothelial protein C receptor (EPCR), and intercellular adhesion molecule 1 (ICAM-1) (13–15). Group B and C (BC) PfEMP1 that contain cysteine-rich interdomain region (CIDR) α2 to α6 domains bind to CD36 and are associated with parasites which cause mild or uncomplicated malaria (16, 17) while the A-type PfEMP1 that contain CIDRα1 domains that bind to EPCR are associated with severe childhood malaria (14, 18–21). However, finding a correlation between expression of PfEMP1 that bind to ICAM-1 and malaria outcome has been much more challenging, with conflicting results about whether ICAM-1 binding is linked to cerebral malaria (22–25). ICAM-1 binding is mediated by a subset of Duffy binding-like (DBL) domains, the DBLβ domains, which are found in either A-type or BC-type PfEMP1 (26–29). Recent studies showed that A-type PfEMP1 with an ICAM-1 binding DBLβ domain also contain a neighboring EPCR-binding CIDRα domain and that these dual-binding PfEMP1 are associated with cerebral malaria (22, 30, 31). No such correlation has been made for BC-type PfEMP1 containing a DBLβ domain.

Structural studies have given significant insight into diversity and conservation of receptor binding sites in PfEMP1. Mapping sequence diversity for EPCR-binding CIDRα domains onto the structure of a CIDRα1 domain bound to EPCR reveals that, while the EPCR binding surfaces are highly diverse in sequence, they retain a specific shape and overall chemical properties (18). This allows conservation of ligand binding, despite sequence diversification. This is similar to the CD36-binding CIDRα domains, where the shape and chemistry of the binding site are conserved despite extensive sequence diversity (16). In contrast, a similar analysis shows that DBLβ domains from A-type ICAM-1 binding PfEMP1 are significantly less diverse. Instead, they contain a sequence motif which shows nearly total conservation in residues which directly contact ICAM-1 or that are responsible for the correct fold of the ICAM-1 binding site. However, this sequence motif is absent in DBLβ domains from B- or C-type PfEMP1 that bind ICAM-1 (22). To understand how these BC-type PfEMP1 interact with ICAM-1, we have determined the structure of the DBLβ domain of the B-type IT4var13 PfEMP1 in complex with the N-terminal domains of ICAM-1, revealing divergent, but similar, modes of ICAM-1 binding across the PfEMP1.

## Results

### ICAM-1 Binding Domains from BC-Type and A-Type PfEMP1 Are Evolutionarily Distinct.

**Fig. 1.**
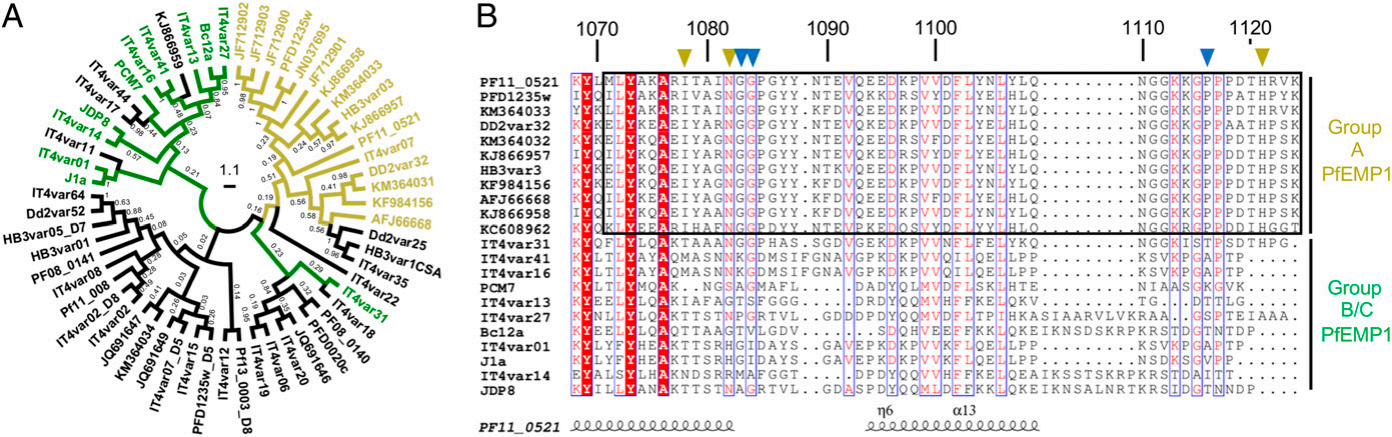
Group A and group BC PfEMP1 do not share a conserved ICAM-1 binding site. (*A*) Maximum likelihood tree based on 59 DBLβ domain sequences. The tree is drawn to scale, with branch lengths measured in the number of substitutions per site. Numbers on the branches show bootstrap values. DBLβ domains from A-type ICAM-1 binding PfEMP1 are highlighted in yellow, DBLβ domains from BC-type ICAM-1 binding PfEMP1 in green, and DBLβ domains shown not to bind to ICAM-1 are in black (see also *SI Appendix*, Table S1). (*B*) Multiple sequence alignment of DBLβ domains from ICAM-1 binding PfEMP1. Numbers show positions in the PF11_0521 DBLβ domain. The box highlights the A-type ICAM-1 binding site. Yellow triangles are residues critical for direct interaction of A-type PfEMP1 with ICAM-1. Blue triangles are residues important for the conformation of the A-type ICAM-1 binding site.

We first performed an updated phylogenetic analysis of all DBLβ domains that have been tested for the ability to bind to ICAM-1 (*SI Appendix*, Table S1). Since publication of similar analyses, in which the only available B- and C-type PfEMP1 sequences were from the IT4 strain of *P. falciparum* (22, 26, 28), 3 ICAM-1 binding DBLβ domains from non-IT4 group B and C PfEMP1 have been identified (27). Inclusion of these sequences confirmed the clustering of A-type ICAM-1 binding domains observed earlier and showed that ICAM-1 binding domains from group B and C PfEMP1 mostly form a separate, albeit less well-defined, cluster (Fig. 1*A*). This clustering was particularly prominent in the region of the sequence which corresponds to the ICAM-1 binding site of the A-type DBLβ domains, with BC-type DBLβ domains lacking all of the essential features of the A-type binding site (Fig. 1*B*). An exception to this was the DBLβ domain of the B-type PfEMP1 IT4var31, which contained many of the key features of the conserved A-type ICAM-1 binding site, but differed from the consensus sequence at crucial isoleucine and proline residues (I1078 and P1116) (Fig. 1*B*). This suggests that IT4var31 is either an A-type PfEMP1 that has diverged in this region or is an intermediate between A-type and BC-type PfEMP1. With this exception, the differences between ICAM-1 binding DBLβ domains indicate that A-type and BC-type PfEMP1 vary in their ICAM-1 binding sites and potentially also differ in their engagement of the receptor.

### The Structural Basis for ICAM-1 Binding by BC-Type PfEMP1.

To understand these differences in ICAM-1 binding and to allow comparison with the previous structure of an A-type ICAM-1 binding DBLβ domain, we aimed to structurally characterize a BC-type PfEMP1 DBLβ domain bound to ICAM-1. We purified a diverse set of DBLβ domains, either alone or bound to the 2 N-terminal domains of ICAM-1 (ICAM-1^D1D2^) and conducted crystallization trials. To remove any flexible regions that might hinder crystallization, we added proteases to the crystallization drops. The DBLβ domain from IT4var13 formed crystals which diffracted to 2.17 Å resolution (Table 1). We determined its structure by molecular replacement, using a truncated version of the PF11_0521 DBLβ domain (PlasmoDB Gene ID PF3D7_1150400) as a search model (PDB ID code 5MZA) (22). A complex of the same domain bound to ICAM-1^D1D2^ formed crystals in the presence of carboxypeptidase B, and these diffracted to 3.67 Å resolution (Table 1), allowing structure determination using a known structure of ICAM-1^D1D2^ (PDB ID code 1IC1) (32) and a pruned version of the final structure of the IT4var13 DBLβ domain as molecular replacement search models.

**Table 1.** Crystallographic data collection and refinement statistics

	IT4var13 DBLβ	IT4var13 DBLβ-ICAM-1^D1D2^
Data collection		
Space group	P12_1_1	P6_5_22
Unit cell parameters		
a, b, c, Å	67.3, 43.4, 87.1	142.4, 142.4, 224.2
α, β, γ, ^O^	90.0, 103.6, 90.0	90.0, 90.0, 120.0
Resolution, Å	32.61–2.17 (2.21–2.17)	112.11–3.67 (3.73–3.67)
* R*_merge_	0.12 (1.55)	0.24 (1.88)
* R*_pim_	0.07 (0.92)	0.08 (0.63)
*I*/sig*I*	5.30 (1.56)	6.50 (1.02)
CC_1/2_	0.99 (0.30)	0.98 (0.55)
Completeness, %	99.81 (96.85)	100 (100)
Redundancy	4.34 (4.35)	9.83 (9.34)
Wilson B factor, Å^2^	40.58	124.80
No. of unique reflections	26,322 (1,229)	15,394 (758)
Refinement		
* R*_work_/*R*_free_	19.96/22.85	24.03/28.65
No. of atoms		
Protein	3,600	4,899
Ligand	—	56
Water	122	—
B-Factors, Å^2^		
Protein	62.85	160.02
Ligand	—	215.66
Water	48.81	—
rms deviations		
Bond lengths, Å	0.003	0.003
Bond angles, ^O^	0.457	0.701

Values in parentheses are for the highest resolution shell. The dashes indicate no data.

The IT4var13 DBLβ domain adopts the classical DBL domain fold (33), consisting of an α-helical core decorated by extensive loops (*SI Appendix*, Fig. S1*A*). Comparison of the unbound and the ICAM-1^D1D2^-bound DBLβ domains showed little variation in structure upon ligand binding (root mean square [rms] deviation of 1.07 Å over backbone Cα), with variation predominantly located in loops (residues 805 to 809, 833 to 840, and 1107 to 1117) (*SI Appendix*, Fig. S1*B*).

**Fig. 2.**
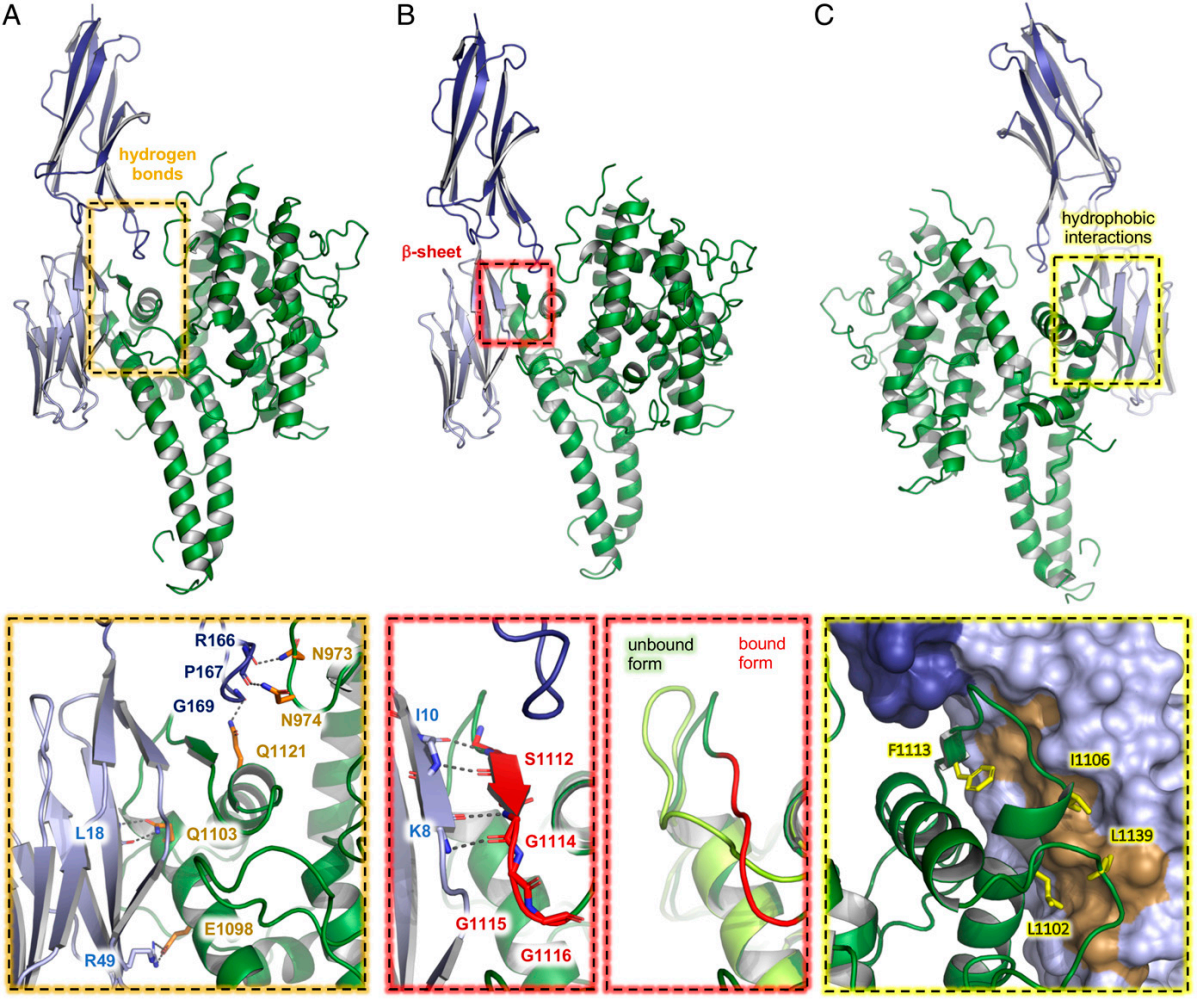
The structural basis for ICAM-1 binding by group B PfEMP1. Front views of the DBLβ domain of IT4var13 (green) bound to ICAM-1^D1D2^ (D1 light blue, D2 dark blue). Dashed boxes highlight the sites that contact ICAM-1 through (*A*) side chain-mediated hydrogen bonds or (*B*) a β-sheet augmentation. A third dashed box compares a region of the ICAM-1–bound and unbound conformations of the IT4var13 DBLβ domain. (*C*) Back view of the IT4var13 DBLβ–ICAM-1^D1D2^ complex. The dashed box highlights the site of hydrophobic contacts between the DBLβ domain and ICAM-1^D1D2^ and the residues in the DBLβ domain that contact a hydrophobic patch on the surface of ICAM-1^D1^ (dark yellow).

The IT4var13 DBLβ domain interacts with domain 1 and 2 of ICAM-1 through a complex binding site, the location of which is compatible with a previous study (28), and binding is mediated by 3 types of interaction (Fig. 2 and *SI Appendix*, Fig. S1*C* and Table S2). A major part of the interface is mediated by hydrogen bonds, contributed by DBLβ domain side chains from a central loop (residues 973 to 976) and helices in the C-terminal third (subdomain 3) of the domain (residues 1098 to 1121) (Fig. 2*A*). All of these DBLβ side chains targets the backbone of ICAM-1, with the exception of glutamate 1098, which forms hydrogen bonds with arginine 49 in ICAM-1^D1^. Secondly, a loop from the DBLβ domain (residues 1107 to 1117) interacts through backbone–backbone hydrogen bonds with ICAM-1^D1^ to add an antiparallel β-strand to the A′GFC β-sheet of ICAM-1^D1^ (Fig. 2*B*). Such β-strand augmentation is a common motif in protein–protein interactions (34) but has not been observed in other PfEMP1–receptor interactions. In the unbound DBLβ domain, this loop adopts a different conformation which is stabilized through crystal contacts (Fig. 1*B* and *SI Appendix*, Fig. S1*D*), indicating that it is flexible and becomes stabilized upon interaction with ICAM-1. This is analogous to a different intrinsically disordered loop that becomes ordered to form part of the ICAM-1 binding site of A-type PfEMP1 (22). Finally, the interface is stabilized by a cluster of hydrophobic residues found on an elongated helix of the DBLβ domain and 2 subsequent loops (residues 1102 to 1139) (Fig. 2*C*). This cluster complements a hydrophobic patch on ICAM-1^D1^ which has previously been identified as important for ICAM-1 binding by B-type PfEMP1 (27, 35, 36).

### Limited Sequence Conservation of the BC-Type ICAM-1 Binding Site.

**Fig. 3.**
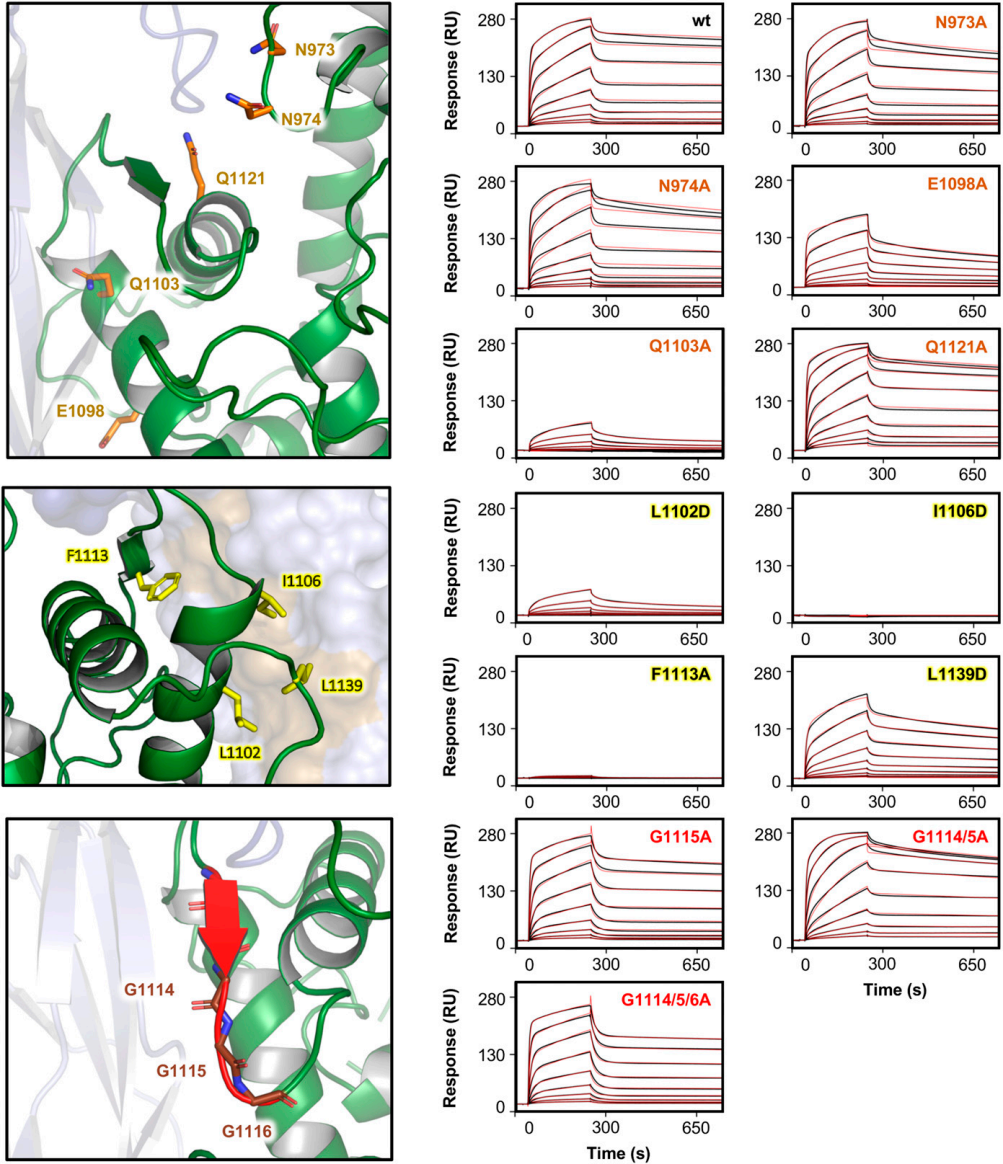
Mutational analysis of the ICAM-1 binding site from group B PfEMP1. The different sites of contact between the IT4var13 DBLβ domain and ICAM-1^D1D2^ and the residues involved in the binding site are shown. For SPR measurements, 2-fold dilution series from 250 nM to 0.9 nM of wild-type (wt) or mutant IT4var13 DBLβ were injected over ICAM-1^D1D5^-Fc immobilized on a Protein A sensor chip. Sensorgrams show the data (black lines) and the fit of a 2-state reaction model (red lines).

We next assessed the role of different residues for binding of the IT4var13 DBLβ domain to ICAM-1 by surface plasmon resonance (SPR). Since the interaction involves a flexible loop that becomes ordered upon ICAM-1 binding, we first used an SPR-based assay to determine if the binding event involves a time-dependent conformational rearrangement. In such a case, the amount of stable complex formed can depend on the association time, with longer association times required to allow the second step of a 2-step binding process to take place, resulting in larger quantities of stable complex and slower dissociation rates (37). Indeed, the binding of IT4var13 DBLβ to ICAM-1 showed such behavior, with the dissociation rate decreasing with an increase in association time, suggesting reordering of components of the binding site (*SI Appendix*, Fig. S2). For this reason, we fitted subsequent SPR data to a 2-state binding model which better describes such interactions involving a conformational change, determining an affinity of 1.22 nM for IT4var13 DBLβ to ICAM-1 (Fig. 3 and *SI Appendix*, Table S3).

We next analyzed through mutagenesis which features of the binding site are essential for interaction with ICAM-1, aiming to identify conserved markers of ICAM-1 binding among BC-type DBLβ domains. We mutated each of the side chains in the IT4var13 DBLβ domain that directly contact ICAM-1 through hydrogen bonds or nonpolar interactions and tested them for binding to ICAM-1^D1D5^-Fc by SPR (Fig. 3). We found that only 1 (Q1103) of the 5 side chains that hydrogen bond with ICAM-1 played a major role in binding, with the Q1103A mutation causing a 200-fold decrease in affinity (Fig. 3 and *SI Appendix*, Table S3). In contrast, mutation of 3 of the 4 hydrophobic residues that contact ICAM-1 (L1102, I1106, and F1113) reduced the interaction affinity by more than 200-fold (Fig. 3 and *SI Appendix*, Table S3), highlighting the importance of this hydrophobic patch for ICAM-1 binding. None of these mutations disrupted the structure of the DBLβ domain, as determined by circular dichroism (CD) spectroscopy and thermal melt experiments (*SI Appendix*, Fig. S3). We also mutated 3 glycines that form part of the loop that interacts with ICAM-1 by β-strand augmentation to test whether they confer flexibility that allows the loop to adopt its bound conformation, but these mutations did not affect the interaction (Fig. 3 and *SI Appendix*, Table S3).

**Fig. 4.**
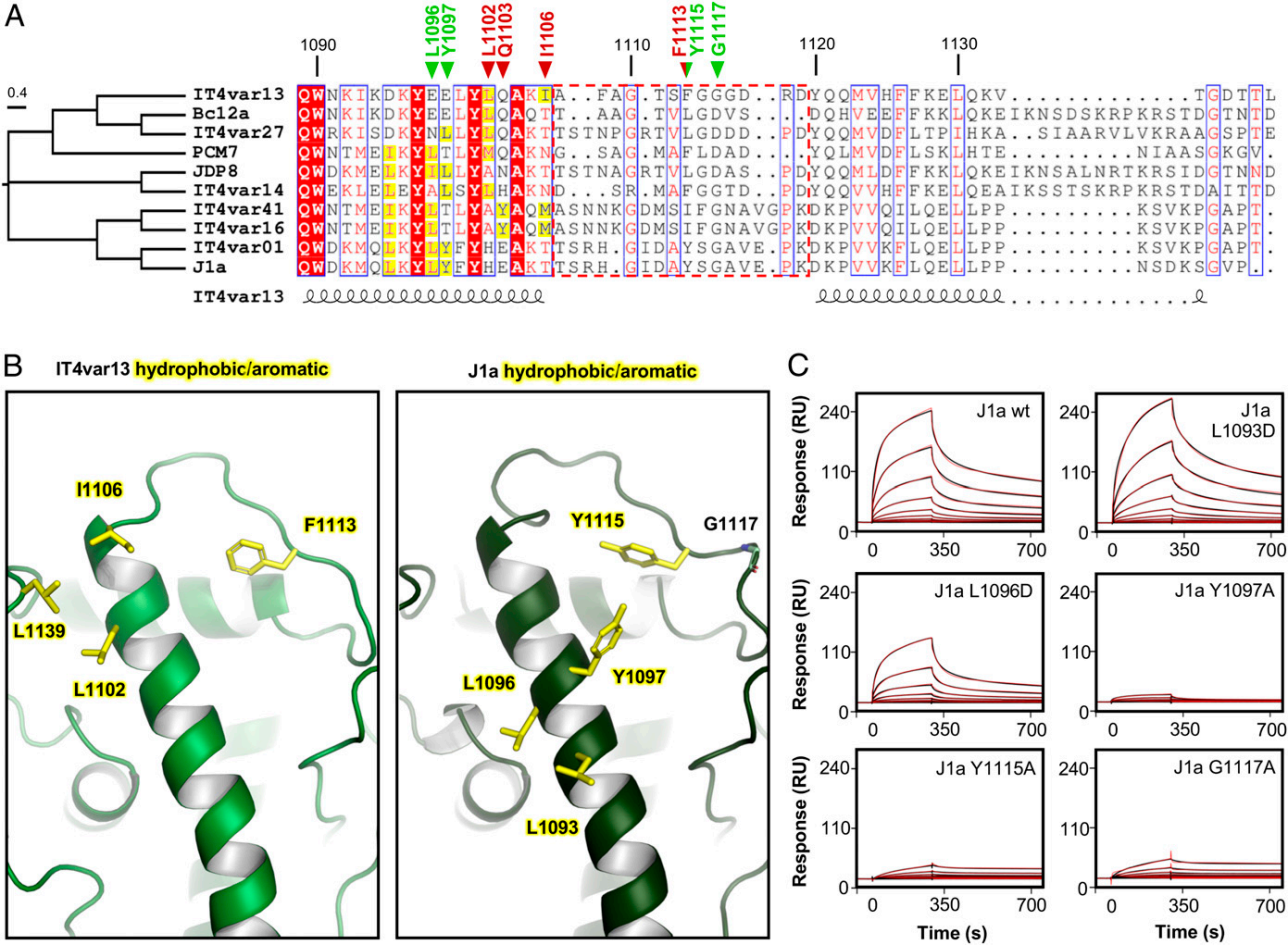
Limited conservation of the ICAM-1 binding site among group BC PfEMP1. (*A*) Multiple sequence alignment of DBLβ domains known to bind ICAM-1. Numbers indicate positions in the IT4var13 DBLβ domain. Residues critical for the IT4var13 DBLβ–ICAM-1 interaction are marked with red triangles while those critical for ICAM-1 binding by J1a DBLβ are marked with green triangles. A half green and half red triangle marks a residue important for both IT4var13 and J1a DBLβ to bind ICAM-1. The flexible loop is indicated with a dashed red box. Surface-exposed hydrophobic and aromatic residues along the helix that forms part of the ICAM-1 binding site are highlighted in yellow. (*B*) Positions of hydrophobic and aromatic residues in the ICAM-1 binding site of IT4var13 DBLβ and equivalent positions in a homology model of the J1a DBLβ domain. (*C*) Binding of J1a DBLβ domain and its mutants to ICAM-1. Wild-type and mutants were injected in a 2-fold dilution series from 500 nM to 0.9 M over ICAM-1^D1D5^-Fc immobilized on a Protein A sensor chip. Sensorgrams show the data (black lines) and the fit of a 2-state reaction model (red lines).

To determine whether these binding properties are conserved, we analyzed the degree of conservation of the interacting residues among the known ICAM-1 binding DBLβ domains from other BC-type PfEMP1, excluding the outlier IT4var31. Sequence alignment revealed limited conservation in sequence and chemical properties among these residues, with hydrophobic residues exchanged for polar residues and vice versa (Fig. 4*A*). The only notable exception was a phenylalanine, F1113, which forms part of the hydrophobic patch that binds to ICAM-1 and is an aromatic or hydrophobic residue in all domains analyzed. Despite the lack of sequence identity, we noted that all ICAM-1 binding DBLβ domains displays surface-exposed hydrophobic and aromatic residues along the top third of the elongated α-helix that forms part of the binding site, albeit in positions that varied within a range of 4 helical turns (Fig. 4 *A* and *B*). To test whether these residues are equivalent in function to the hydrophobic patch in IT4var13, we introduced mutations into the DBLβ domain of the C-type PfEMP1 J1a (27) and tested their effect on binding to ICAM-1 by SPR. With the exception of L1093D, these mutations reduced binding and affinity by 2- to 20-fold, which was not due to the disruption of the overall fold of the domain, as verified by CD spectroscopy and thermal melt experiments (Fig. 4*C* and *SI Appendix*, Fig. S4 and Table S4). Of these residues, Y1115 in J1a is equivalent in position to F1113 in IT4var13 and performed an equivalently important role in binding. Y1097 and L1096 in J1a form a hydrophobic patch which appeared functionally equivalent to the patch formed by residues L1102 and I1106 in IT4var13, despite being found in a different region of the sequence and a different location on the helix. This use of similar binding features, contributed by different regions of the domains, illustrates significant plasticity in how the BC-type DBLβ bind to ICAM-1.

The flexible loop that binds ICAM-1 by β-strand augmentation in IT4var13 DBLβ is also present in other BC type PfEMP1 (Fig. 4*A*). To test whether these use a similar mechanism of β-strand addition to bind ICAM-1, we once again assessed the dependence of dissociation rate on association time (*SI Appendix*, Fig. S2). For each of the tested DBLβ domains from BC-type PfEMP1 (IT4var13, J1a, and Bc12a), the specific dissociation rate after binding to ICAM-1 decreased with an increase in association time, indicating that the interaction involves reordering of components of the binding site (*SI Appendix*, Fig. S2). We observed the same effect for DBLβ domains from A-type PfEMP1 (Pf11_0521 and PFD1235w), in which a different flexible loop becomes ordered during ICAM-1 binding (22), but not for CIDRα domains (HB3var3 and IT4var20) that bind to their receptor EPCR without any apparent structural rearrangement (18).

While the flexible loop varies significantly in length between B- and C-type ICAM-1 binding DBLβ domains, most variants contain a glycine that follows the conserved hydrophobic or aromatic residue (Fig. 4*A*). While these glycine residues had no significance for ICAM-1 binding in IT4var13 DBLβ (Fig. 3), they might be necessary for longer variants of the loop to adopt the ICAM-1 bound conformation. To test this, we mutated this glycine in the J1a DBLβ and found a 7-fold reduced binding level and 1.5-fold reduced affinity, highlighting the importance of flexibility of this elongated loop (Fig. 4*C*).

In summary, the ICAM-1 binding site of BC-type PfEMP1 is defined by the overall chemical nature of key elements, such as a surface-exposed hydrophobic patch and a flexible loop that becomes ordered upon ICAM-1 binding. However, the position, sequence and length of these elements varies significantly, in stark contrast to the highly conserved binding site of A-type PfEMP1.

### Comparison of ICAM-1 Binding by DBLβ Domains from A- and BC-Type PfEMP1.

**Fig. 5.**
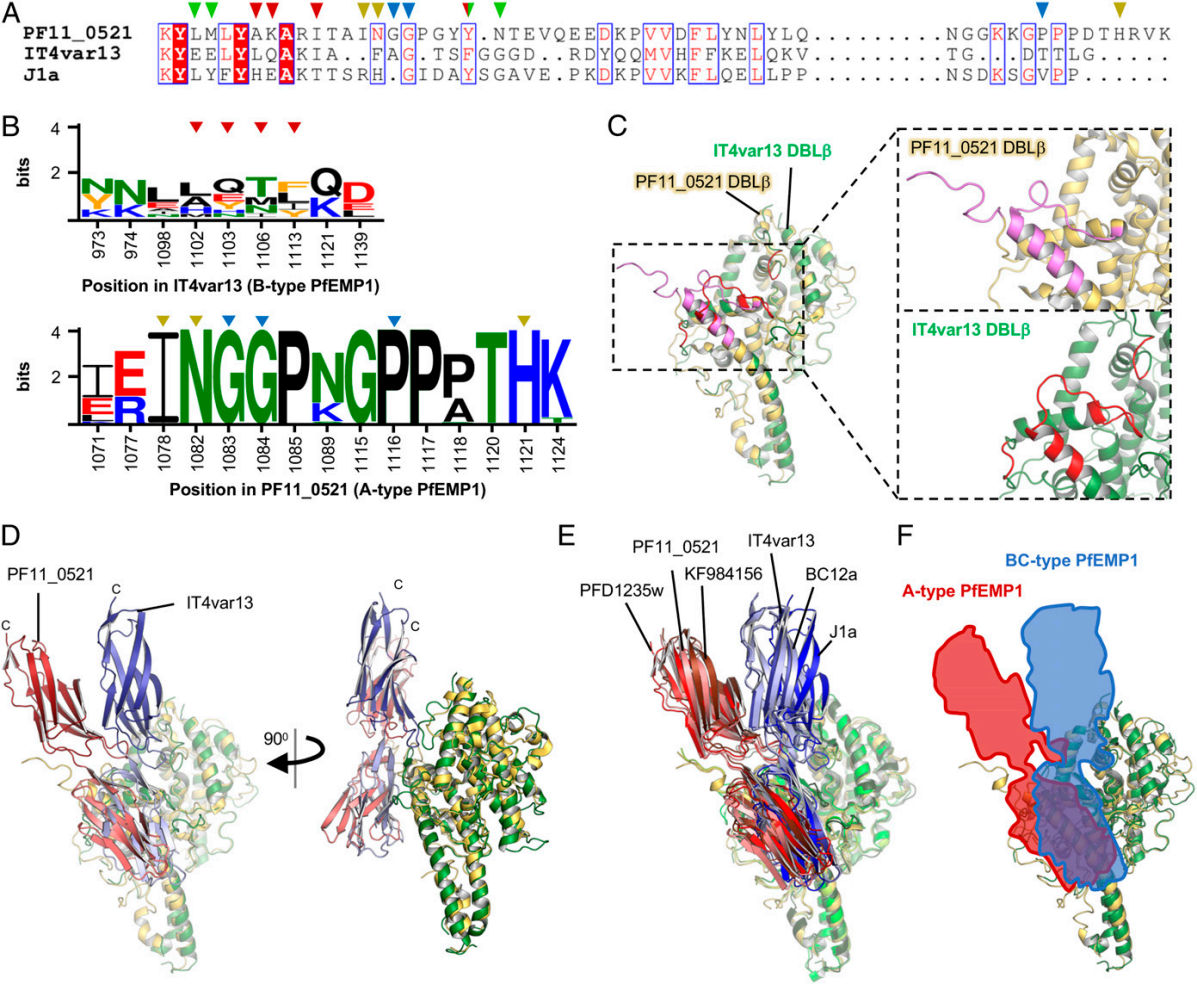
A-type and BC-type PfEMP1 bind ICAM-1 at different angles. (*A*) Sequence alignment of PF11_0521, IT4var13, and J1a DBLβ based on the alignment shown in Figs. 1 and 4. The residues shown by mutagenesis to be important for ICAM-1 binding are marked by triangles. Red triangles mark residues important in IT4var13, green in J1a, half red and half green in both IT4var13 and J1a, yellow to interact with ICAM-1 in PF11_0521, and blue to stabilize the structure of the binding site in PF11_0521. (*B*) Sequence logo showing all residues involved in ICAM-1 binding of A- or BC-type PfEMP1, based on 10 BC-type or 145 A-type DBLβ domains known or predicted to bind ICAM-1. Numbering is based on the IT4var13 and PF11_0521 sequences. Red triangles mark residues of IT4var13 that directly interact with ICAM-1. Yellow triangles mark residues of PF11_0521 that directly interact with ICAM-1. Blue triangles mark residues important for the conformation of the ICAM-1 binding site in PF11_0521. (*C*) Superposition of the DBLβ domains from PF11_0521 and IT4var13 in their ICAM-1 bound conformation. The ICAM-1 binding sites for PF11_0521 (pink) and IT4var13 (red) DBLβ are indicated. The *Inset* shows a magnification of the ICAM-1 binding sites of the individual domains. (*D*) Overlay of the ICAM-1 bound complexes of IT4var13 DBLβ and PF11_0521 DBLβ, with the DBLβ domains superimposed. The C terminus of ICAM-1^D1D2^ is indicated. (*E*) Models for complexes of 3 DBLβ domains from A-type PFEMP1 or BC-type PfEMP1 bound to ICAM-1, superimposed on the DBLβ domain. The models were derived from docking with HADDOCK, followed by filtering using SEC-SAXS data. (*F*) Schematic representation of the overall binding angles between ICAM-1 and DBLβ domains from A-type (red) and BC-type (blue) PfEMP1.

Phylogenetic analyses of ICAM-1 binding DBLβ domains (Fig. 1) and the significant variation in sequence conservation between the binding sites from A-type and BC-type PfEMP1 (Fig. 5*A*) suggests that 2 variants of ICAM-1 binding sites have evolved and exist in parallel, a property so far unique among PfEMP1–receptor interactions. We therefore asked what the specific differences between these 2 types of binding sites are and whether these have any functional consequences for ICAM-1 binding.

The A-type PfEMP1 PF11_0521 and the B-type PfEMP1 IT4var13 have ICAM-1 binding sites in similar locations. However, the B-type binding site is more compact, and the overall structure of the DBLβ domain in this region is more similar to that of previously characterized non-ICAM-1 binding domains (Fig. 5*B* and *SI Appendix*, Fig. S5) (38). In contrast, the A-type binding site protrudes from this framework through an elongated α-helix and a loop that, in the ICAM-1 bound conformation, projects away from the domain. Interestingly, this also results in a different binding angle for ICAM-1 for these 2 domain types. An overlay of the 2 structures, based on the DBLβ domain, shows that, while domain 1 of ICAM-1 is in a similar position in both structures, the protruding A-type ICAM-1 binding site fixes the C terminus of domain 2 at an angle that is ∼30° different from the position that it adopts, relative to the DBLβ domain, when bound to the B-type IT4var13 (Fig. 5*D*).

To test whether this difference in angle is a general property of A- and BC-type PfEMP1, we produced 2 additional ICAM-1 binding DBLβ domains from each of the A-type (PFD1235w and KF984156) and BC-type (Bc12a and J1a) PfEMP1. We then collected solution small angle X-ray scattering (SAXS) data for all 6 domains, either alone or in complex with ICAM-1^D1D2^ (*SI Appendix*, Table S5) and used these data to guide molecular docking experiments, with the crystal structures of ICAM-1^D1D2^, the PF11_0521 and IT4var13 DBLβ domain structures, and homology models of the other domains as input for HADDOCK (39, 40). The models of the DBLβ ICAM-1^D1D2^ complexes obtained after SAXS filtering (*SI Appendix*, Fig. S6*A*) showed that this method reproduces the complexes observed in the crystal structures with high precision (root mean square deviation over backbone Cα of 0.83 Å for PF11_0521 DBLβ-ICAM^D1D2^ and 1.47 Å for IT4var13 DBLβ-ICAM^D1D2^) (*SI Appendix*, Fig. S6*B*). More importantly, it showed that the resulting models fit into 2 distinct classes with respect to the ICAM-1 angle, with all of the A-type DBLβ domains in the same class as PF11_0521 while the BC-type DBLβ domains group with IT4var13 (Fig. 5 *E* and *F*). Ab initio envelopes calculated from the SAXS data further supported this observation and indicated that the differences are not due to significantly different shapes of the DBLβ domains themselves (*SI Appendix*, Fig. S7). Taken together, these data show that the structural variations between the 2 types of ICAM-1 binding site result in different binding angles for this receptor and that this difference is consistent between A-type and BC-type PfEMP1.

## Discussion

The PfEMP1 DBLβ domains that bind to ICAM-1 are unusual in that they segregate into 2 evolutionarily distinct clusters, the A- and BC-types (Fig. 1) (22, 26). In this study, we compare our structure of a BC-type ICAM-1 binding DBLβ domain with the previously determined structure of an A-type domain (22), revealing that both contain a globally similar ICAM-1 binding site, in a similar location. However, these 2 domains differ in the conformation of the ICAM-1 binding region, the degree of sequence conservation of the binding site, and the angle at which ICAM-1 is bound relative to the DBLβ domain. This raises the question of why the parasite uses 2 similar and yet distinct types of binding sites to interact with the same receptor.

The BC-type DBLβ domains and other PfEMP1 domains share the overall chemical nature of their interactions with their ligands. The core of their binding site is a hydrophobic cluster, which is complemented by hydrogen bonds contributed by both side chains and backbone groups. These features are similar to those by which DBLβ domains from A-type PfEMP1 interact with ICAM-1 (22). A similar picture is also seen in CIDRα domains that interact with EPCR and CD36 (16, 18), which bind their receptors through hydrophobic or aromatic residues that either protrude from the domain or form a cavity that accepts hydrophobic residues from the receptor. Therefore, in all of the structurally characterized PfEMP1–receptor interactions, a hydrophobic patch lies at the heart of the binding site, and mutation of these hydrophobic residues has a major effect on the overall affinity, with changes in both association and dissociation rates (*SI Appendix*, Tables S3 and S4) (16, 18, 22). In addition, in all 4 cases, adjacent residues mediate hydrogen bonds that stabilize the interaction and generate increased surface complementarity with the receptor. This conservation of a hydrophobic core, together with larger complementary hydrophilic surfaces, allows the formation of stable PfEMP1–receptor complexes, enabling infected red blood cells to withstand the forces of blood flow during cytoadhesion and to evade splenic clearance.

Despite these shared features, there are significant differences in the degree of sequence conservation between ICAM-1 binding sites of the A- and BC-type DBLβ domains. The residues through which BC-type PfEMP1 bind to ICAM-1 are highly variable in sequence, and the relative positions of interacting residues and the length of the loop that forms a β-sheet addition upon ICAM-1 binding differ between domain variants. This is conceptually similar to CIDRα domains that bind EPCR or CD36 (16, 18), which also retain the overall shape and the chemistry of their binding sites, while at the same time diversifying in sequence. In marked contrast, in the A-type PfEMP1 that bind ICAM-1, all residues that are critical for direct interaction with the receptor, or the positioning of these side chains, are absolutely conserved (22).

So, which is more common: a binding site with a high level of sequence variation or one in which interacting residues are conserved? In the context of the pressure to diversify to maintain the capacity for immune evasion, theory would predict the former. Indeed, PfEMP1 containing the highly variable EPCR, CD36, and ICAM-1 binding sites are abundant in the PfEMP1 repertoire of parasite genomes from reference strains or field isolates. For example, in the 3D7 genome, 84% of PfEMP1 contain domains that bind CD36 and 11% contain EPCR-binders while, in the IT4 genome, 11% of PfEMP1 contain BC-type ICAM-1 binding DBLβ domains (41). In contrast, A-type ICAM-1 binding sites occur at an average frequency of 1 per genome (∼1.3% of PfEMP1) (42). Therefore, a highly conserved binding site is the exception rather than the rule.

This raises the question of why the parasite has evolved and maintained 2 similar and yet distinct classes of binding sites to interact with the same receptor. In particular, the highly conserved A-type ICAM-1 binding site would appear disadvantageous under immune pressure. Indeed, antibodies that block EPCR binding by CIDRα1 domains have very limited cross-inhibitory activity against other variants of this domain (43). In contrast, antibodies that target the A-type binding site can broadly cross-inhibit ICAM-1 binding, and children in malaria-endemic regions rapidly acquire such antibodies (22, 44–46). A potential explanation for retention of the A-type binding site, despite its being the target of such a cross-inhibitory immune response, is that it confers a specific advantage compared to the more variable BC-type binding site. Indeed, some studies suggest that A-type PfEMP1 are preferentially expressed in individuals with limited immunity and that antibodies against these PfEMP1 are acquired first (47, 48), indicating that they can confer an early survival advantage for the parasite.

A possible reason why parasites have evolved both A- and BC-type ICAM-1 binding domains is that, while these domains bind to ICAM-1 equally well (22, 27, 30, 49–51), they may differently influence the ability of neighboring domains to bind additional receptors. Previous studies have shown that all PfEMP1 that contain a DBLβ domain with an A-type ICAM-1 binding site also contain an adjacent CIDRα1 domain with an EPCR-binding site and that these PfEMP1 can bind simultaneously to both receptors (22, 30). Such dual binding is not limited to A-type PfEMP1 since some BC-type PfEMP1 containing an ICAM-1 binding domain also have a CD36-binding domain (8, 15, 29), and ICAM-1 and CD36 have been shown to cooperate to enhance the binding of infected erythrocytes to microvascular cells (15, 52). However, in A-type PfEMP1, dual binding to ICAM-1 and EPCR has been shown to specifically enhance binding of infected erythrocytes to endothelial cells under physiologically higher shear stresses (22, 50), which offers a distinct advantage to the parasite, especially for cytoadherence to brain endothelial cells where CD36 is absent.

The ability of a PfEMP1 to bind simultaneously to 2 membrane-bound receptors will depend on its architecture and how it presents these binding sites. The majority of PfEMP1 are thought to be rigid, elongated proteins (49, 53), constraining the conformation in which they can interact with 2 membrane-bound receptors simultaneously. As the distance between the host cell membrane and the PfEMP1 binding site is ∼66 Å for CD36 and ∼32 Å for EPCR, it is tempting to speculate that the A-type and BC-type DBLβ domains have evolved to bind ICAM-1 at different angles in order to allow the neighboring CIDRα domain better to interact with either EPCR or CD36 at different heights from the host endothelial membrane. Future studies and structural insights into full-length PfEMP1 will be needed to test this hypothesis.

In summary, our characterization of the ICAM-1 binding site of the BC type PfEMP1 supports the view that the majority of PfEMP1 utilize a binding site that is highly variable in sequence but conserved in shape and chemistry, allowing these proteins to retain receptor-binding capacity while evading immune detection. This also highlights the unusual conservation of the A-type ICAM-1 binding site, reinforcing the view that it is a suitable target for the development of antidisease malaria vaccines.

## Materials and Methods

### Protein Expression and Purification.

The constructs for the PF11_0521, PFD1235w, KF984156, BC12a, and J1a DBLβ domains were described previously (22, 26, 27). Constructs comprised the following amino acids of the respective PfEMP1: 728 to 1214 of PF11_0521, 739 to 1221 of PFD1235w, 546 to 1036 of KF984156, 813 to 1273 of Bc12a, 739 to 1195 of J1a, and 733 to 1202 of IT4var13. All constructs had an N-terminal hexa-histidine tag, followed by a Tobacco Etch Virus (TEV) protease cleavage site in the pET15b expression vector. They were expressed in Shuffle 3030 *Escherichia coli* (New England Biolabs) at 25 °C for 16 h. The DBLβ domains were purified by affinity chromatography using nickel-nitrilotriacetic acid agarose (Ni-NTA) (Qiagen), followed by size exclusion chromatography using a HiLoad Superdex 75 16/60 column (GE Healthcare). Mutants of IT4var13 and J1a DBLβ were made using the QuikChange site-directed mutagenesis protocol (Agilent Technologies) and expressed and purified as described for wild-type protein.

Constructs for ICAM-1^D1D5^-Fc and His-tagged ICAM-1^D1D2^ were described previously (49, 54). ICAM-1^D1D5^-Fc comprises amino acids 1 to 480 of human ICAM-1, fused to the Fc part of human IgG in a mammalian expression vector (54). ICAM-1^D1D2^ comprises amino acids 28 to 212 of human ICAM-1, fused to a C-terminal hex-histidine tag in the pHLsec expression vector (55). All constructs were transiently expressed as secreted proteins in HEK293F cells (Life Technologies). For ICAM-1^D1D2^, kifunensine (Cayman Chemical) was added to the medium during transfection to a final concentration of 1.5 µM. Seven days after transfection, the cell culture supernatants were harvested and sterile-filtered. ICAM^D1D2^ was then purified by affinity chromatography using Ni-NTA affinity (Qiagen), and ICAM-1^D1D5^-Fc was purified by affinity chromatography using a HiTrap Protein A HP column (GE Healthcare). Both proteins were further purified by size exclusion chromatography using a HiLoad Superdex 75 16/60 column (GE Healthcare). The HB3var and IT4var20 CIDRα domains and EPCR were produced as described previously (18).

### Crystallization.

For crystallization, Ni-NTA purified IT4var13 DBLβ was further purified by size exclusion chromatography using a HiLoad Superdex 75 16/60 column (GE Healthcare) into 10 mM 4-(2-hydroxyethyl)-1-piperazineethanesulfonic acid (Hepes), 150 mM NaCl, pH 7.2. For complex formation, IT4var13 DBLβ was mixed with a 1.5-fold molar excess of ICAM-1^D1D2^ and purified as described for IT4var13 DBLβ. Fractions containing pure IT4var13 DBLβ or IT4var13 DBLβ-ICAM^D1D2^ complex were pooled and concentrated to 23 mg/mL. Crystals were grown by vapor diffusion in sitting drops by mixing 100 nL of protein solution with 100 nL of well solution. To remove flexible loops, Carboxypeptidase B (Sigma Aldrich), Chymotrypsin (Sigma Aldrich), or Endoproteinase GluC (New England Biolabs) was added to the crystallization drop in a 100:1 (protein:protease) molar ratio. Crystals of IT4var13 DBLβ grew at 291 K in the presence or absence of protease in conditions from the JCSG+ screen (Molecular Dimensions) containing 0.2 M Magnesium chloride hexahydrate, 0.1 M BIS-Tris, pH 5.5, and 25% (wt/vol) polyethylene glycol (PEG) 3350. For cryoprotection, crystals were transferred into well solution containing 25% glycerol and were flash-frozen in liquid nitrogen. Crystals of the IT4var13 DBLβ-ICAM^D1D2^ complex grew only in the presence of Carboxypeptidase B at 291 K in conditions from the ProPlex screen (Molecular Dimensions) containing 0.1 M Tris, pH 8.0, and 25% (vol/vol) methoxypolyethylene glycol 350 (PEG 350 MME). The crystals were cryoprotected by transfer into well solution containing 0.1 M Tris, pH 8.0, and 32% (vol/vol) PEG 350 MME and were flash-frozen in liquid nitrogen.

### Data Collection, Phasing, and Refinement.

Data for crystals of IT4var13 DBLβ were collected at beamline IO4-1 (Diamond Light Source) using X-rays at a wavelength of 0.92 Å and a Pilatus 6M-F detector (Dectris, Baden-Daettwil, Switzerland). Data for crystals of the IT4var13 DBLβ-ICAM^D1D2^ complex were collected at beamline IO3 (Diamond Light Source) using X-rays at a wavelength of 1.0 Å and an Eiger2 × 16M detector (Dectris, Baden-Daettwil, Switzerland). Both datasets were processed using the XIA2/DIALS pipeline (56) in the CCP4i2software suite (57, 58) for indexing and scaling.

The structure of IT4var13 DBLβ was solved by molecular replacement with Phaser (59) using a search model consisting of a poly-alanine model of the PF11_0521 DBLβ domain (PDB ID code 5MZA) (22) in which helices in subdomain 3 were shortened and all loops were removed. Molecular replacement found 1 copy of the search model in the asymmetric unit, and the remaining parts of IT4var13 DBLβ were built through iterative cycles of model building in Coot (60) and refinement in Phenix (61).

The structure of the IT4var13 DBLβ-ICAM^D1D2^ complex was solved by molecular replacement with Phaser using pruned poly-alanine models of the IT4var13 DBLβ domain and ICAM-1^D1D2^ (PDB ID code 1IC1) (32) as search models. To allow for flexibility between domain 1 and domain 2 of ICAM-1^D1D2^, the domains were used as separate search models. Molecular replacement found 1 copy of the complex in the asymmetric unit, and the remaining parts were built through iterative cycles of model building in Coot and refinement in Phenix. The structures were refined to final Ramachandran statistics of 95.6% residues in the favored regions, 4.4% in the allowed regions, and no residues in the disallowed regions for IT4var13 DBLβ; and 95.0% residues in the favored regions, 5.0% in the allowed regions, and no residues in the disallowed regions for the IT4var13 DBLβ-ICAM^D1D2^ complex. The coordinate and structure factor data are deposited in the protein data bank (PDB) under the accession codes 6S8T (IT4var13 DBLβ) and 6S8U (IT4var13 DBLβ-ICAM^D1D2^). All figures showing structures were prepared with PyMol (Schroedinger LLC).

### Surface Plasmon Resonance Spectroscopy.

SPR experiments were conducted on a Biacore T200 instrument (GE Healthcare). All proteins were purified by size exclusion chromatography, and only fractions with >95% purity were used for SPR. To test binding of DBLβ wild-type and mutant proteins to ICAM-1, ICAM-1^D1D5^-Fc was immobilized to 850 response units (RUs) for IT4var13 DBLβ proteins or 490 RUs for J1a DBLβ proteins, on a CM5 chip (GE Healthcare) precoupled with Protein A (Sigma Aldrich). All DBLβ proteins were buffer-exchanged into 20 mM Hepes, 300 mM NaCl, 0.005% Tween 20, pH 7.2, and concentration series (0.9 nM to 250 nM for IT4var13 DBLβ and 0.9 nM to 500 nM for J1a DBLβ) were injected over the chip at 30 µL/min, with a 240-s association time and 600-s (for IT4var13 DBLβ) or 720-s (for J1a DBLβ) dissociation time. After each run, the chip was regenerated by injecting 10 mM Glycine, pH 1.7, for 120 s at 10 µL/min.

To test the effect of association time on dissociation rate, ICAM-1^D1D5^-Fc was immobilized to 490 RUs on a CM5 as described above, and biotinylated EPCR was immobilized to 340 RUs on a CAP chip using the Biotin Capture Kit (GE Healthcare). DBLβ or CIDRα domains were then injected over the chip at a fixed concentration of 3 µM for 60 s, 120 s, 180 s, or 240 s, with a dissociation time of 300 s. After each run, the chip was regenerated by injecting CAPture Kit regeneration solution (GE Healthcare) (for CIDRα binding to EPCR) or 10 mM Glycine, pH 1.7 (for DBLβ binding to ICAM-1) for 120 s at 10 µL/min.

All data were analyzed using BIAevaluation software 2.0.3 (GE Healthcare). Kinetic values were determined by globally fitting the curves into a 2-state reaction model. All SPR experiments were performed in duplicate, and curves shown are representatives of these measurements.

### Size Exclusion Chromatography Coupled Small Angle X-Ray Scattering.

All size exclusion chromatography coupled (SEC)-SAXS experiments were carried out at the B21 beamline (Diamond Light Source), using X-rays at a wavelength of 0.99 Å and an Eiger 4M detector (Dectris, Baden-Daettwil, Switzerland) with a detector-sample distance of 4.014 m. For data collection, samples were concentrated and injected at 20 °C over a Superdex 200 Increase 3.2/300 column equilibrated with 20 mM Hepes, 150 mM NaCl, pH 7.2, with a 2-s exposure for each frame. The data were processed using the ScÅtter (62) and ATSAS (63) software suites, and buffer frames were averaged and subtracted from averaged frames corresponding to peak fractions. The radius of gyration (R_g_) was calculated by Guinier analysis using AutoRg in PRIMUS (64). The distance distribution function P(r) and the maximum particle diameter Dmax were determined using GNOM (65). To generate volumetric representations of envelopes, 20 ab initio bead models were generated using DAMMIF (66). These models were than averaged with DAMAVER (67), followed by refinement against the original data using DAMMIN (68). The resulting bead models were then used to calculate envelopes with Situs. Crystal structures of the PF11_0521 and IT4var13 DBLβ domains, either alone or in complex with ICAM-1^D1D2^, as well as models of the other DBLβ domains, alone or in complex with ICAM-1^D1D2^, were fitted into the envelopes using Chimera (69). All figures showing envelopes were made with PyMol (Schroedinger LLC).

### Circular Dichroism Spectroscopy.

All CD spectra were recorded on a J-815 Spectropolarimeter (Jasco), connected to a Peltier temperature control unit. All samples were dialyzed against 100 mM sodium phosphate buffer, 200 mM NaF, pH 7.2, and adjusted to 0.3 mg/mL. CD spectra were recorded at 20 °C using a cell with a path length of 1 mM at wavelengths between 195 nm and 250 nm, and spectra recorded for buffer were subtracted from these measurements. For thermal melt experiments, spectra were recorded between 200 nm and 250 nm, and, between each measurement, the temperature was increased by 0.5 °C increments.

### Molecular Modeling and Docking Experiments.

Homology models of DBLβ domains used for molecular docking experiments were generated with SwissModel (70), using structures of the ICAM-1 bound form of PF11_0521 DBLβ (for KF984156 DBLβ and PFD1235w DBLβ) or IT4var13 DBLβ (for Bc12a DBLβ and J1a DBLβ) as templates. To assemble complexes of DBLβ domain and ICAM-1^D1D2^ by molecular docking, HADDOCK (39) was used. For this, the structures or models of the DBLβ domains and ICAM-1^D1D2^ were used as input for the HADDOCK web server. DBLβ residues identified to be critical for interaction of A-type PfEMP1 (22) or BC-type PfEMP1 (this study) with ICAM-1, and ICAM-1 residues known to be important for the PfEMP1-ICAM interaction (22, 27), were defined as active residues while passive residues were chosen automatically. Other parameters were as default. Models from each docking experiment were filtered against solution scattering data from SEC-SAXS, as described by Karaca and Bonvin (40), and the top scoring model was selected as the final docked complex.

### Phylogenetic Analysis.

For phylogenetic analysis of ICAM-1 binding and nonbinding DBLβ domains, 59 sequences of DBLβ domains with known ICAM-1 binding phenotype (*SI Appendix*, Table S1) were aligned using MUSCLE (71). The evolutionary history was inferred using the Maximum Likelihood method based on the Whelan and Goldman + Freq. model (72) using Mega 7 (73). The tree with the highest log likelihood (−25861.52) is shown. The percentage of trees in which the associated taxa clustered together is shown next to the branches. Initial tree(s) for the heuristic search were obtained automatically by applying Neighbor-Join and BioNJ algorithms to a matrix of pairwise distances estimated using a JTT model, and then selecting the topology with superior log likelihood value. A discrete Gamma distribution was used to model evolutionary rate differences among sites (5 categories; +G, parameter = 1.1732). The rate variation model allowed for some sites to be evolutionarily invariable ([+I], 14.79% sites). The tree is drawn to scale, with branch lengths measured in the number of substitutions per site. All positions with less than 95% site coverage were eliminated. That is, fewer than 5% alignment gaps, missing data, and ambiguous bases were allowed at any position. There were 360 positions in the final dataset. The tree was visualized using FigTree version 1.4.3. A sequence logo for residues involved in ICAM-1 binding by A-type or BC-type PfEMP1 was generated using WebLogo 3 (74), based on 145 protein sequences containing the A-type ICAM-1 binding motif (22) or 10 sequences of BC-type PfEMP1 known to bind ICAM-1 (27, 29, 75).

### Data Availability.

Data for the structures reported here have been deposited in the PDB under the accession codes 6S8T and 6S8U.

## Supplementary Material

Supplementary File
