# Supplementary material for: Structural insights into diverse modes of ICAM-1 binding by Plasmodium falciparum-infected erythrocytes
Source: Proc Natl Acad Sci U S A. 2019 Sep 16;116(40):20124–34. doi: 10.1073/pnas.1911900116 (PMC6778195; doi:10.1073/pnas.1911900116)
Supplement: Supplementary File [file pnas.1911900116.sapp.pdf]

Supplementary Information

Structural insights into diverse modes of ICAM-1 binding by *Plasmodium falciparum*-infected erythrocytes.

Frank Lennartz, Cameron Smith, Alister G. Craig and Matthew K. Higgins

www.pnas.org/cgi/doi/10.1073/pnas.1911900116

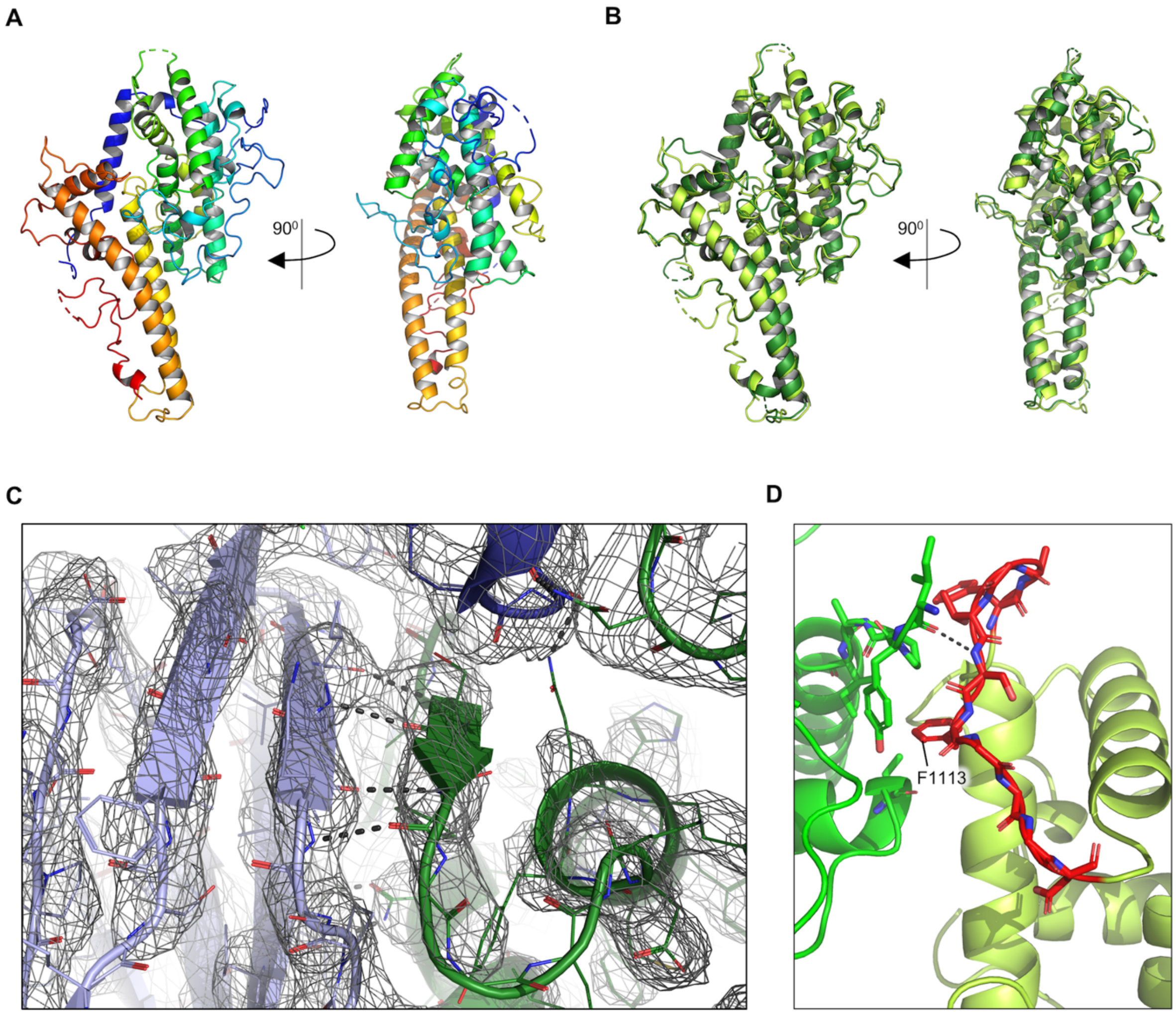

Fig. S1 – Comparison of the structure of IT4var13 DBLβ in the ICAM-1^{D1D2} bound and unbound forms.

(A) Structure of the IT4var13 DBLβ domain in its ICAM-1 bound form. The domain is color coded from the N-terminus (blue) to the C-terminus (red). **(B)** Superposition of the structure of the IT4var13 DBLβ in its unbound (bright green) and ICAM-1 bound (dark green) forms. **(C)** Sample electron density (feature-enhanced 2FoFc map) for the ICAM-1 binding site in the IT4var13 DBLβ-ICAM-1^{D1D2} complex, contoured at a sigma level of 1.0. Dashed lines indicate hydrogen bonds. **(D)** Crystal contacts around the flexible loop of the ICAM-1 binding site (red) between symmetry mates (green and bright green) in the crystals of the unbound IT4var13 DBLβ domain. Dashed lines indicate hydrogen bonds. For orientation, the residue F1113 is indicated.

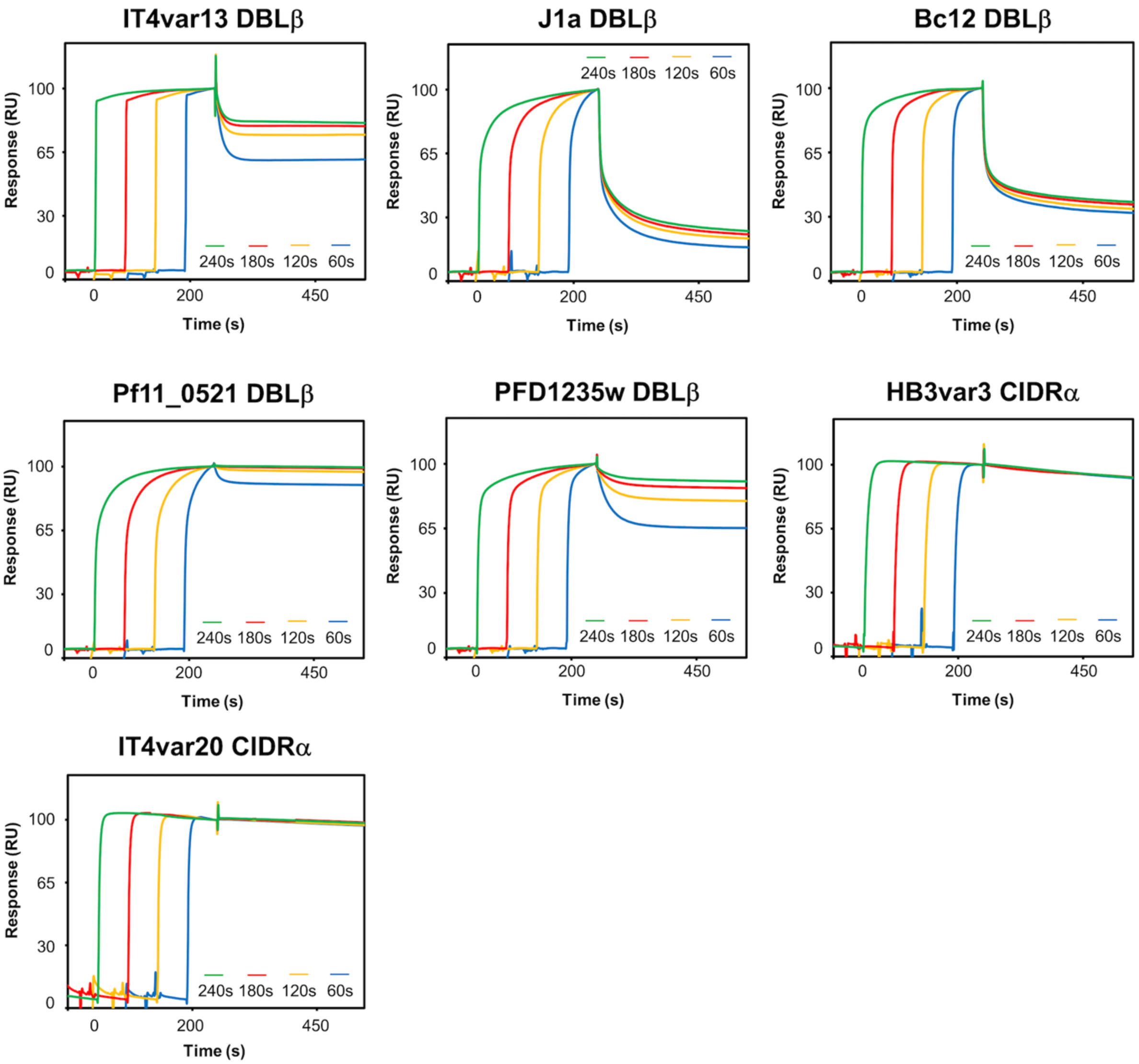

Fig. S2 – SPR analysis of potential conformational changes upon receptor binding for DBLβ and CIDRα domains.

The domains were injected over their respective receptors at a fixed concentration of 3μM. For each injection, the association time was varied in 60s intervals between 60s and 240s. The data were then superimposed using the end of the injection as fixed time point and normalized by setting the signal at the start of the injection to 0 and at the stop of the injection to 100, to monitor how the dissociation rate changes with the length of association time. DBLβ domains were injected over ICAM-1^{D1D5}–Fc immobilized on a CM5 sensor chip pre-coupled with Protein A. CIDRα domains were injected over biotinylated EPCR immobilized on a CAP sensor chip.

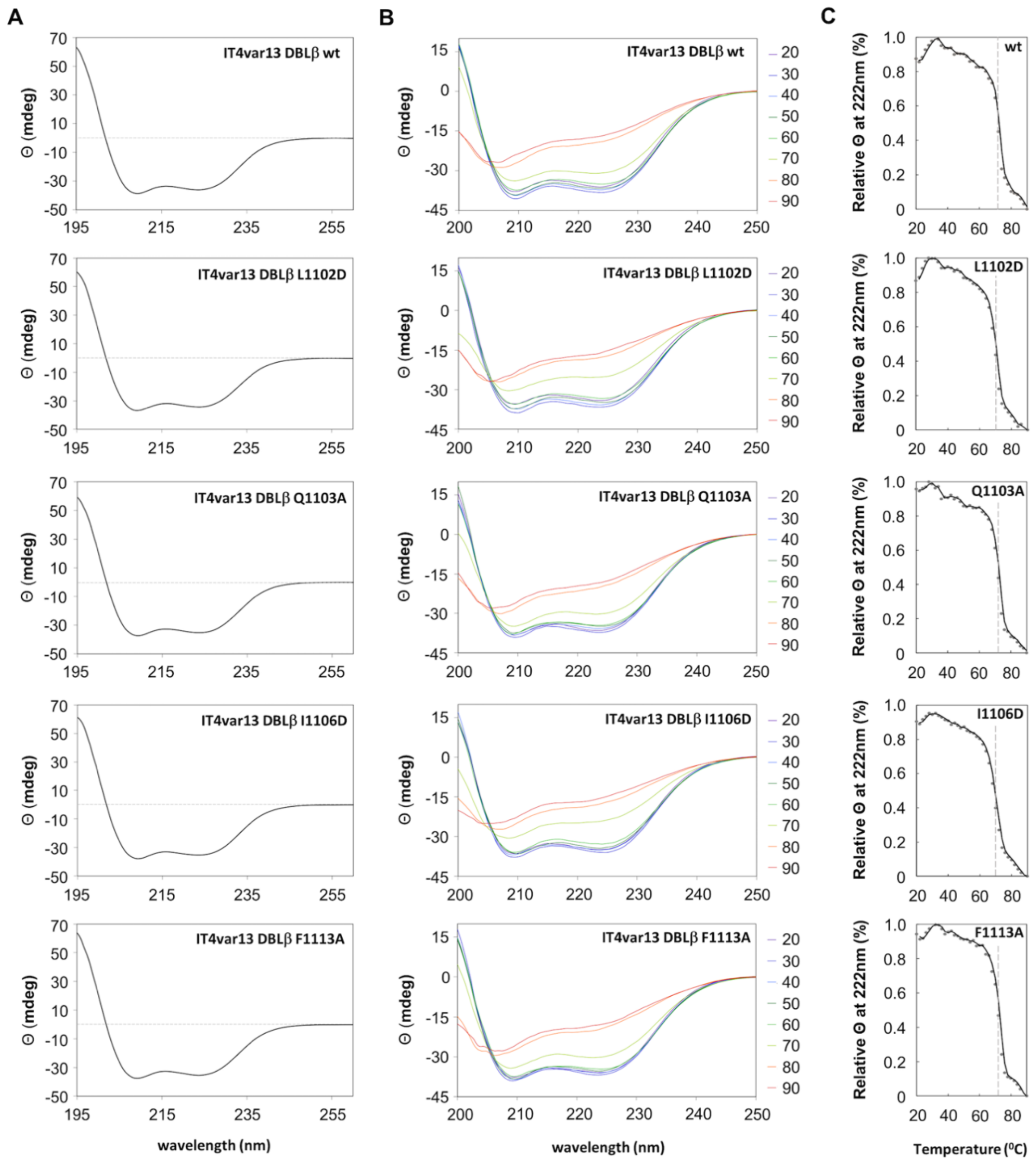

Fig. S3 – CD spectroscopy and melting curves for IT4var13 DBLβ wild type and mutants **(A)** Secondary structure analysis of the IT4var13 DBLβ wild-type (wt) domain and mutants. CD spectra were recorded at 20°C between wavelengths of 195 nm and 260 nm. For each sample, four measurements were averaged and corrected for buffer absorption. **(B)** Thermal melt analysis of IT4var13 DBLβ wild type and mutant domains. CD spectra were recorded between 200 nm and 250 nm and after each measurement the temperature was raised by 0.5°C. Shown are spectra collected at 10°C intervals between 20°C and 90°C. **(C)** Thermal denaturation curve for IT4var13 DBLβ wild type and mutants. The ellipticity at a fixed wavelength of 222 nm was measured between 20°C and 90°C. After each measurement the temperature was increased by 0.5°C. A dashed line indicates the melting point.

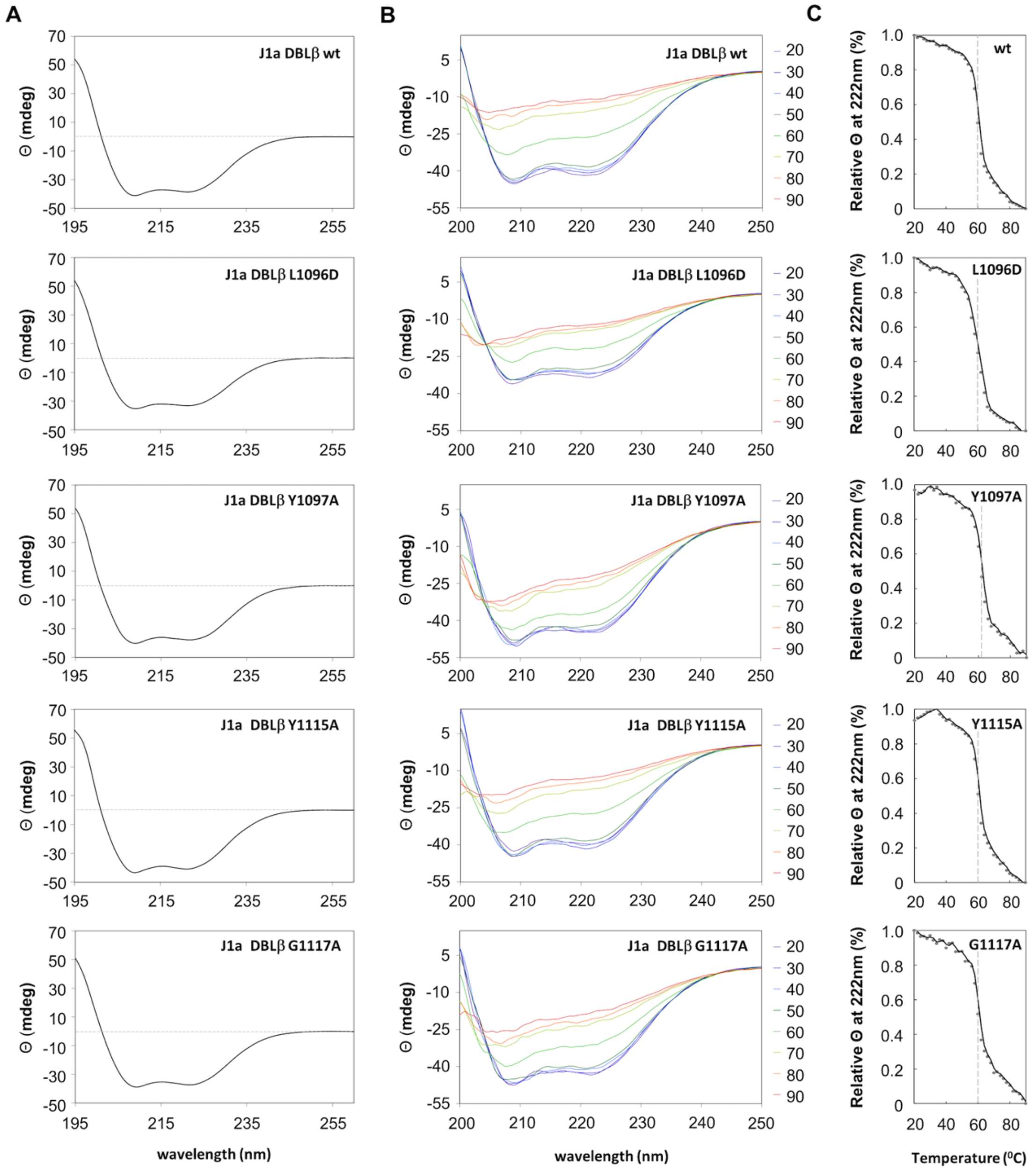

Fig. S4 – CD spectroscopy and melting curves for J1a DBLβ wild type and mutants

(A) Secondary structure analysis of the J1a DBLβ wild-type (wt) domain and mutants. CD spectra were recorded at 20°C between wavelengths of 195 nm and 260 nm. For each sample, four measurements were averaged and corrected for buffer absorption. **(B)** Thermal melt analysis of J1a DBLβ wild type and mutant domains. CD spectra were recorded between 200 nm and 250 nm and after each measurement the temperature was raised by 0.5°C. Shown are spectra for 10°C intervals between 20°C and 90°C. **(C)** Thermal denaturation curve for J1a DBLβ wild type and mutants. The ellipticity at a fixed wavelength of 222 nm was measured between 20°C and 90°C. After each measurement, the temperature was increased by 0.5°C. A dashed line indicates the melting point.

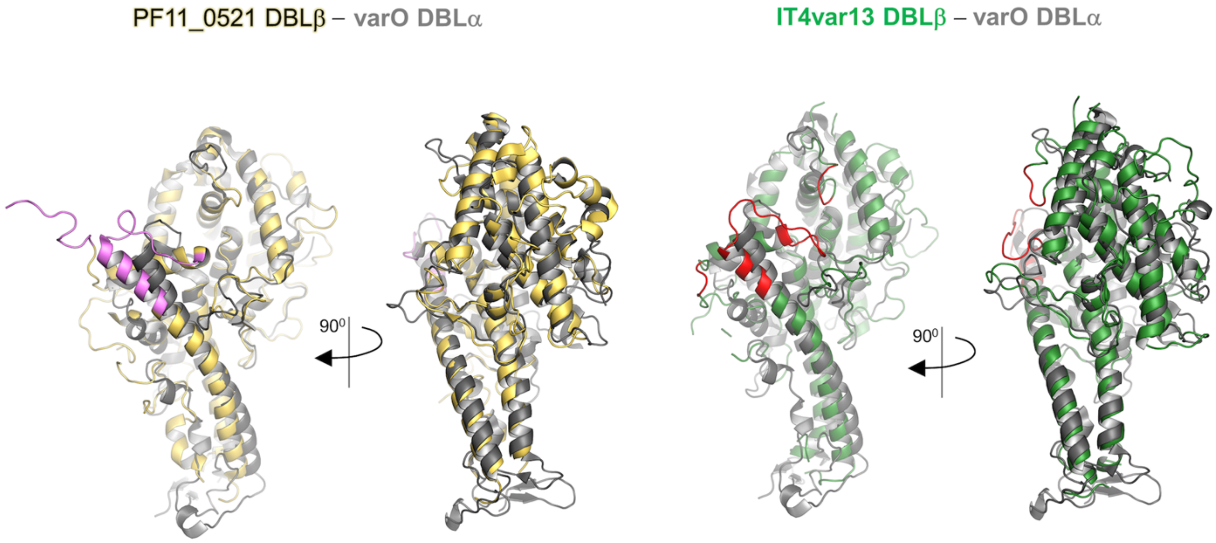

Fig. S5 - Superposition of the DBLβ domains from IT4var13 and PF11_0521 PfEMP1 onto the DBLα domain from the varO PfEMP1.

IT4var13 DBLβ is colored in green and the ICAM-1 binding site of this domain is colored in red. PF11_0521 DBLβ is colored in bright green and the ICAM-1 binding site of this domain is colored in pink. The varO DBLα domain (PDB 2XU0) (1) is colored in grey. The superposition was made using PyMol.

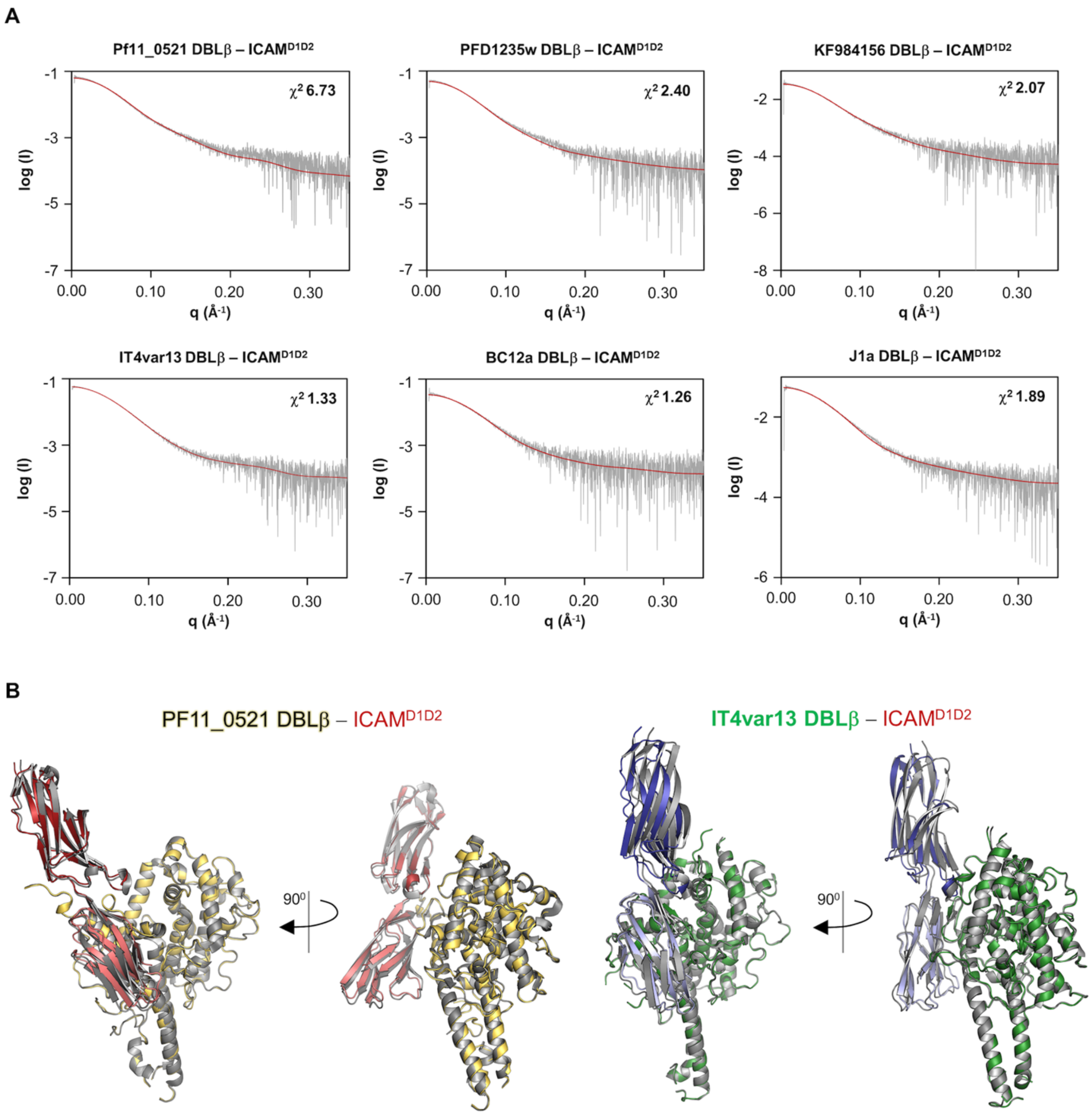

Fig. S6. Crysol fits for DBLβ-ICAM-1^{D1D2} models generated by HADDOCK and comparison with the crystal structures.

(A) Crysol fits between SAXS data and the top models for DBLβ –ICAM-1^{D1D2} complexes selected after SAXS filtering. **(B)** The models for the PF11_0521 DBLβ-ICAM-1^{D1D2} (DBLβ: dark yellow; ICAM-1^{D1D2}: red) and the IT4var13 DBLβ – ICAM-1^{D1D2} (DBLβ: green; ICAM-1^{D1D2}: blue) complexes derived from HADDOCK after SAXS-filtering are superimposed over the crystal structures of the respective complexes (grey).

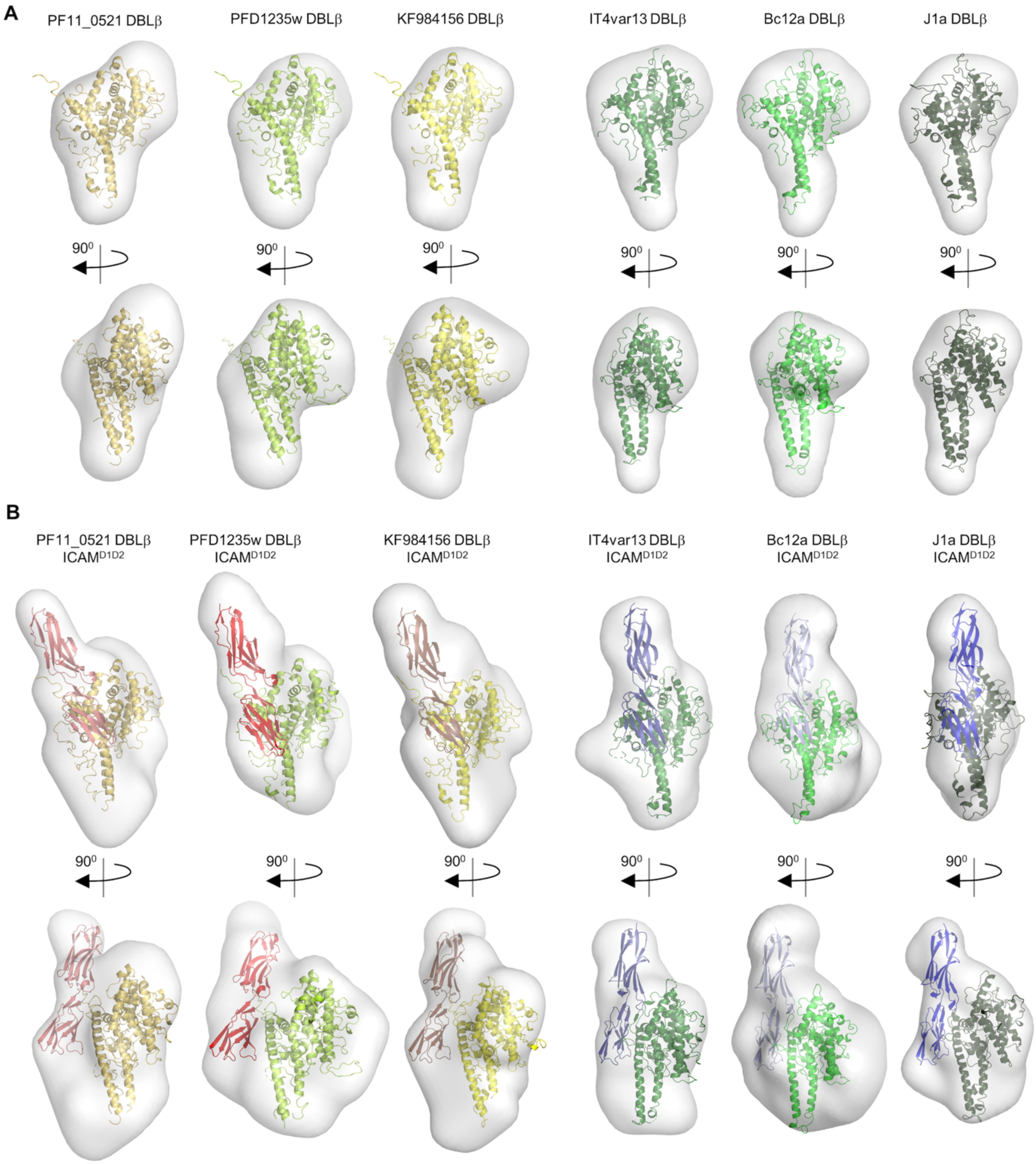

Fig. S7 – SAXS analysis of DBLβ-ICAM-1^{D1D2} complexes.
Ab initio envelopes calculated from SAXS data for DBLβ domains from group A and group BC PfEMP1, either **(A)** alone or **(B)** in complex with ICAM-1^{D1D2}. The models generated by SwissModel for the DBLβ domains alone or by HADDOCK for the DBLβ domains bound to ICAM-1^{D1D2} were docked into the envelopes using Chimera.

Table S1 Sequences used for phylogenetic analyses

PfEMP1	GenBank accession number	ICAM-1 binding phenotype shown in
IT4var01	AAO67411	(2, 3)
IT4var02	AAQ73925	(2, 3)
IT4var02_D8	AAQ73925	(2)
IT4var06	ABM88781	(2, 3)
IT4var07	ABM88782	(4)
IT4var07_D5	ABM88782	(2, 3)
IT4var08	ABM88783	(2, 3)
IT4var11	ETW15085	(2)
IT4var12	AAB06961	(2, 3)
IT4var13	ABM88750	(2, 3, 5)
IT4var14	AAD03351	(2, 3)
IT4var15	AAA75398	(2, 3)
IT4var16	AAS89259	(2, 3, 5)
IT4var17	ABM88751	(2, 3)
IT4var18	ABM88752	(2, 3)
IT4var19	ABM88753	(2, 3)
IT4var20	AAA75397	(2, 3)
IT4var22	ABM88754	(2, 3)
IT4var27	ABM88759	(2, 3, 5)
IT4var31	AAF18980	(2, 3, 5)
IT4var35	ADK78857	(2, 3)
IT4var41	ABM88768	(2, 3, 5)
IT4var44	ABM88769	(2)
IT4var64	ABM88780	(2)
Bc12a	AVI24062	(6)
J1a	AVI24063	(6)

PCM7	AVI24066	(6)
JDP8	AAK49742	(7)
PF08_0140	XP_001349512	(8)
PF08_0141	XP_001349513	(8)
PF11_0521	XP_001348176	(8, 9)
PF11_008	XP_001347692	(8, 10)
PF13_0003_D8	XP_001349740	(8)
PFD1235w	XP_002808895	(8, 10)
PFD1235w_D5	XP_002808895	(8, 10)
PFD0020c	EWC90558	(8)
HB3var01	KOB61483	(8)
HB3var03	KOB63865	(8)
HB3var1CSA	KOB61785	(8)
HB3var05_D7	KOB63865	(8)
Dd2var25	KOB84767	(8)
DD2var32	KOB85388	(8, 10)
Dd2var52	ABM88780	(8)
JF712902	AEQ26010	(8, 10)
JF712903	AEA86277	(8, 10)
JF712900	JF712900	(8, 10)
JF712901	JF712901	(8, 10)
KF984156	AIX97102	(8)
KM364031	AIY25809	(8)
KM364034	AIY25811	(8)
KM364033	AIY25810	(8)
KJ866957	AIX97164	(8)
KJ866958	AIX97165	(8)
KJ866959	AIX97166	(8)
JQ691646	AFJ66676	(8)

JQ691647	AFJ66677	(8)
JQ691649	AFJ66679	(8)
AFJ66668	AFJ66668	(8)
JN037695	AEI26313	(8)

Table S2 – List of contacts between IT4var13DBLβ and ICAM-1^{D1D2}.

Colors correspond to those used in Fig. 2 of the main text.

IT4var13 DBLβ		ICAM-1^{D1D2}		
**Residue**	**Group**	**Residue**	**Group**	**Type of Interaction**
**Asn973**	Sidechain NH_2	**Arg166**	Backbone O	Hydrogen bond
**Asn974**	Sidechain NH_2	**Arg167**	Backbone O	Hydrogen bond
**Glu1098**	Sidechain O	**Arg49**	Sidechain NH_2	Hydrogen bond
**Leu1102**	Sidechain	**Leu44**	Sidechain	Hydrophobic
**Leu1102**	Sidechain	**Val51**	Sidechain	Hydrophobic
**Gln1103**	Sidechain O	**Leu18**	Backbone N	Hydrogen bond
**Gln1103**	Sidechain NH_2	**Leu18**	Backbone O	Hydrogen bond
**Ile1106**	Sidechain	**Leu18**	Sidechain	Hydrophobic
**Ser1112**	Backbone N	**Ile10**	Backbone O	Hydrogen bond
**Ser1112**	Backbone O	**Ile10**	Backbone N	Hydrogen bond
**Phe1113**	Sidechain	**Val9**	Sidechain	Hydrophobic
**Phe1113**	Sidechain	**Leu11**	Sidechain	Hydrophobic
**Phe1113**	Sidechain	**Val17**	Sidechain	Hydrophobic
**Gly1114**	Backbone N	**Lys8**	Backbone O	Hydrogen bond
**Gly1114**	Backbone O	**Lys8**	Backbone N	Hydrogen bond
**Gln1121**	Sidechain NH_2	**Gly169**	Backbone O	Hydrogen bond
**Leu1139**	Sidechain	**Leu42**	Sidechain	Hydrophobic

Table S3 – Kinetic parameters derived from SPR for binding of ICAM-1^{D1D5} to IT4var13 DBLβ wild type and mutants

Interaction	k_a1 ($\times10^5$ M^{-1} s^{-1})	k_d1 (10^{-2} s^{-1})	k_a2 ($\times10^{-2}$ s^{-1})	k_d2 (10^{-4} s^{-1})	K_D (nM)
ICAM^{D1D5} - IT4var13 DBLβ wt	6.47	7.76	1.13	1.17	1.22
ICAM^{D1D5} - IT4var13 DBLβ N973A	36.40	60.80	1.66	3.24	3.22
ICAM^{D1D5} - IT4var13 DBLβ N974A	64.00	99.22	1.53	3.02	2.99
ICAM^{D1D5} - IT4var13 DBLβ E1098A	4.26	9.37	1.21	7.82	13.40
ICAM^{D1D5} - IT4var13 DBLβ L1102D	0.21	2.46	0.54	10.91	196.00
ICAM^{D1D5} - IT4var13 DBLβ Q1103A	0.09	1.47	0.43	8.98	259.00
ICAM^{D1D5} - IT4var13 DBLβ I1106D	0.80	87.74	0.28	452.40	10300.00
ICAM^{D1D5} - IT4var13 DBLβ F1113A	0.05	3.71	0.11	10.31	3620.00
ICAM^{D1D5} - IT4var13 DBLβ Q1121A	13.60	8.49	1.49	2.11	0.86
ICAM^{D1D5} - IT4var13 DBLβ L1139D	3.19	6.07	1.18	5.89	9.06
ICAM^{D1D5} - IT4var13 DBLβ G1115A	8.28	8.37	0.56	1.25	2.21
ICAM^{D1D5} - IT4var13 DBLβ G1114/5A	630.00	685.00	3.07	3.12	1.09
ICAM^{D1D5} - IT4var13 DBLβ G1114/5/6A	7.96	7.54	0.43	0.85	1.86

Table S4 - Kinetic parameters derived from SPR for binding of ICAM-1^{D1D5} to J1a DBLβ wild type and mutants

Interaction	k_a1 ($x10^4$ M^{-1} s^{-1})	k_d1 (10^{-2} s^{-1})	k_a2 ($x10^{-3}$ s^{-1})	k_d2 (10^{-4} s^{-1})	K_D (nM)
ICAM^{D1D5} - J1a DBLβ wt	1.75	2.54	4.26	9.40	262.00
ICAM^{D1D5} - J1a DBLβ L1093D	1.07	1.07	3.54	10.64	232.00
ICAM^{D1D5} - J1a DBLβ L1096D	0.74	2.55	3.20	10.81	866.00
ICAM^{D1D5} - J1a DBLβ Y1097A	0.08	3.45	3.46	4.74	5140.00
ICAM^{D1D5} - J1a DBLβ Y1115A	0.16	3.73	7.93	1.71	498.00
ICAM^{D1D5} - J1a DBLβ G1117A	4.27	44.15	4.48	1.51	336.00

Table S5 - Parameters derived from solution scattering data.

	R_g (nm)	D_{max} (nm)	V_{porod} (nm^3)	Mr_{exp} (kDa)	Mr_{app} (kDa)
			**DBLβ domain alone**		
**PF11_0521 DBLβ**	27.30	94.12	84.0	49.80	57.20
**PFD1235w DBLβ**	26.80	86.43	85.0	50.00	56.90
**KF984156 DBLβ**	28.00	88.07	86.9	51.12	57.90
**IT4var13 DBLβ**	26.00	89.35	85.8	50.47	55.60
**Bc12a DBLβ**	26.00	88.64	78.6	46.26	54.60
**J1a DBLβ**	26.40	85.86	79.0	47.00	53.60
			**DBLβ-ICAM^{D1D2}**		
**PF11_0521 DBLβ – ICAM-1^{D1D2}**	35.11	126.10	140.0	89.4	82.00
**PFD1235w DBLβ – ICAM-1^{D1D2}**	35.21	128.07	132.0	90.0	82.00
**KF984156 DBLβ – ICAM-1^{D1D2}**	35.33	127.74	139.0	94.2	82.00
**IT4var13 DBLβ – ICAM-1^{D1D2}**	32.40	110.49	133.0	86.3	82.00
**Bc12a DBLβ – ICAM-1^{D1D2}**	33.74	113.41	115.0	74.9	80.00
**J1a DBLβ – ICAM-1^{D1D2}**	31.08	109.00	98.8	68.6	80.00

The radius of gyration R_g was determined using AutoRg and the maximum particle diameter D_{max} was calculated with GNOM. The Porod volume V_{porod} was calculated with PRIMUS. The apparent molecular mass Mr_{app} was calculated using the from the size and shape function in version 2.8.3 of the ATSAS suite. Mr_{exp} is the theoretical molecular mass.

Supplemental References

1. Juillerat A, et al. (2011) Structure of a Plasmodium falciparum PfEMP1 rosetting domain reveals a role for the N-terminal segment in heparin-mediated rosette inhibition. *Proc Natl Acad Sci* 108(13):5243–5248.
2. Janes JH, et al. (2011) Investigating the host binding signature on the Plasmodium falciparum PfEMP1 protein family. *PLoS Pathog* 7(5). doi:10.1371/journal.ppat.1002032.
3. Howell DPG, et al. (2008) Mapping a common interaction site used by Plasmodium falciparum Duffy binding-like domains to bind diverse host receptors. *Mol Microbiol* 67(1):78–87.
4. Avril M, Bernabeu M, Benjamin M, Brazier AJ, Smith JD (2016) Interaction between endothelial protein C receptor and intercellular adhesion molecule 1 to mediate binding of plasmodium falciparum-infected erythrocytes to endothelial cells. *MBio* 7(4). doi:10.1128/mBio.00615-16.
5. Brown A, et al. (2013) Molecular architecture of a complex between an adhesion protein from the malaria parasite and intracellular adhesion molecule 1. *J Biol Chem* 288(8):5992–6003.
6. Carrington E, et al. (2018) In silico guided reconstruction and analysis of ICAM-1-binding var genes from Plasmodium falciparum. *Sci Rep* 8(1):3282.
7. Chattopadhyay R, Taneja T, Chakrabarti K, Pillai CR, Chitnis CE (2004) Molecular analysis of the cytoadherence phenotype of a Plasmodium falciparum field isolate that binds intercellular adhesion molecule -1. *Mol Biochem Parasitol* 133(2):255–265.
8. Lennartz F, et al. (2017) Structure-Guided Identification of a Family of Dual Receptor-Binding PfEMP1 that Is Associated with Cerebral Malaria. *Cell Host Microbe* 21(3):403–414.
9. Oleinikov A V., et al. (2009) High throughput functional assays of the variant antigen PfEMP1 reveal a single domain in the 3D7 plasmodium falciparum genome that binds ICAM1 with high affinity and is targeted by naturally acquired neutralizing antibodies. *PLoS Pathog* 5(4). doi:10.1371/journal.ppat.1000386.
10. Bengtsson A, et al. (2013) A Novel Domain Cassette Identifies Plasmodium falciparum PfEMP1 Proteins Binding ICAM-1 and Is a Target of Cross-Reactive, Adhesion-Inhibitory Antibodies. *J Immunol* 190(1):240–249.
